# Circular Economy Electrochemistry: Recycling Old Mixed Material Additively Manufactured Sensors into New Electroanalytical Sensing Platforms

**DOI:** 10.1021/acssuschemeng.3c02052

**Published:** 2023-06-07

**Authors:** Robert D. Crapnell, Evelyn Sigley, Rhys J. Williams, Tom Brine, Alejandro Garcia-Miranda Ferrari, Cristiane Kalinke, Bruno C. Janegitz, Juliano A. Bonacin, Craig E. Banks

**Affiliations:** † Faculty of Science and Engineering, Manchester Metropolitan University, Chester Street, Manchester M1 5GD, U.K.; ‡ Institute of Chemistry, University of Campinas (Unicamp), 13083-859 São Paulo, Brazil; § Department of Nature Sciences, Mathematics, and Education, Federal University of São Carlos (UFSCar), 13600-970 Araras, São Paulo, Brazil

**Keywords:** additive manufacturing (3D printing), fused filament fabrication (fused deposition modeling), circular economy electrochemistry, waste plastic, recycling, electroanalysis

## Abstract

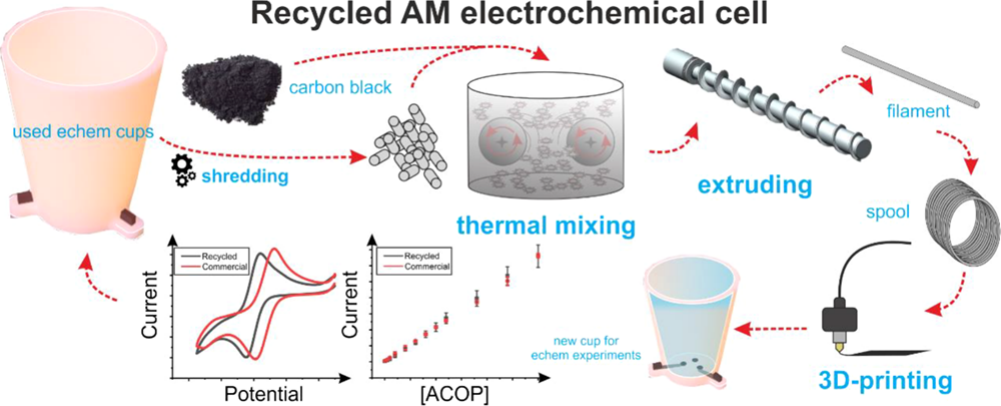

Recycling used mixed material additively manufactured electroanalytical sensors into new 3D-printing filaments (both conductive and non-conductive) for the production of new sensors is reported herein. Additively manufactured (3D-printed) sensing platforms were transformed into a non-conductive filament for fused filament fabrication through four different methodologies (granulation, ball-milling, solvent mixing, and thermal mixing) with thermal mixing producing the best quality filament, as evidenced by the improved dispersion of fillers throughout the composite. Utilizing this thermal mixing methodology, and without supplementation with the virgin polymer, the filament was able to be cycled twice before failure. This was then used to process old sensors into an electrically conductive filament through the addition of carbon black into the thermal mixing process. Both recycled filaments (conductive and non-conductive) were utilized to produce a new electroanalytical sensing platform, which was tested for the cell’s original application of acetaminophen determination. The fully recycled cell matched the electrochemical and electroanalytical performance of the original sensing platform, achieving a sensitivity of 22.4 ± 0.2 μA μM^–1^, a limit of detection of 3.2 ± 0.8 μM, and a recovery value of 95 ± 5% when tested using a real pharmaceutical sample. This study represents a paradigm shift in how sustainability and recycling can be utilized within additively manufactured electrochemistry toward promoting circular economy electrochemistry.

## Abstract 2

Old additively manufactured one-shot sensing platforms are recycled into two new filaments for 3D printing: one conductive and one non-conductive. These filaments are used to print a fully recycled cell, in an identical design, that matches the original cell in electroanalytical performance toward the detection of acetaminophen. This feeds into the concept of an electrochemistry circular economy.

## Introduction

Sustainable development is defined as “development that meets the needs of the present without compromising the ability of future generations to meet their own needs”, and over several decades, it has become an increasingly important topic on the global agenda.^1^ A significant challenge for meeting the United Nations’ sustainable development goals is reducing as far as possible reliance on virgin materials, particularly those derived from non-renewable resources.^2^ While part of this reduction can be achieved through encouraging changes in consumer habits, much of it is expected to be achieved through the reuse of objects where possible or failing that, recycling of their constituent raw materials to make new objects. Together, these two activities form a fundamental backbone of the concept of Circular Economy (CE). While an exact definition of CE has still not been formalized, it is generally understood to refer to an economic system in which raw materials are extracted, used, and reused/recycled repeatedly with zero waste and pollution over their lifetime.^3^ The transition to perfect CEs is for the time being hypothetical, but the ideas nested within the concept are nonetheless gaining increasing traction as design rules for meeting sustainable development goals.^4,5^

Additive manufacturing (AM), also known as 3D printing, refers to a group of manufacturing technologies that are likely to play an important role in developing CEs.^6^ In contrast to well-established subtractive and formative manufacturing methods (e.g., milling or molding), AM technologies form objects by on-demand processing of digital design files into thin cross-sections, which can then be produced using layer-by-layer assembly. The exact means of layer manufacture depends on the technology being used and can include material extrusion from a nozzle, selective sintering of an area of solid powder, or selective photopolymerization of an area of liquid resin.^7^ While AM technologies vary significantly in terms of their fundamental operation, they share many of the same advantages, the most relevant to the CE being their potential for reducing waste and pollution. That is to say, because parts can be produced on-demand using local AM printers, there is both a reduced need to order parts in excess to mitigate uncertain consumer demand and reduced need to ship parts from specialist manufacturers. However, although this does serve to reduce the consumption of raw materials and transport-associated pollution, current practices relating to the end-of-life of AM printed parts often do not align well with CE models. This is because they are usually discarded into conventional waste streams after only a few uses or, in some cases, a single use.

One area in which AM is increasingly being used to produce single-use parts is the field of electrochemistry, with inexpensive fused filament fabrication (FFF) devices being used increasingly for the production of bespoke laboratory parts which might otherwise represent a significant cost.^8^ In particular, polymer composites filled with a conductive filler (commonly carbon black^9^ or graphene^10^) are becoming increasingly popular with electrochemists for the production of cheap equipment,^11^ electrochemical cells,^12,13^ and inventively designed electrodes.^8,14−16^ However, the requirement for cleanliness to achieve repeatable measurements, especially with regard to the fouling of working electrodes,^9^ means that the AM devices used in electrochemistry are often discarded after one use. Simply put, since the typical polymer cells and electrodes made by the AM cost on the order of a few British pence and are known to be affected by cleaning solvents which can dissolve^17^ or be absorbed by the polymer material,^18^ in the short term it is generally easier and more economical to dispose of them. This is not a sustainable practice, and with sustainability becoming an increasingly important theme in scientific research in general,^19^ and electrochemistry specifically,^20^ more work is clearly needed to enable AM in electrochemistry to conform to CE ideals.

In particular, recycling is an attractive means of processing AM electrochemical devices into useful products. More specifically, in contrast to simple reuse, recycling of 3D printed electrodes removes most concerns about fouling, since thermal processing (e.g., melt extrusion) should destroy many organic contaminants, and otherwise distribute them away from the surface regions important for electrochemical processes. Furthermore, it makes better use of the design flexibility inherent to AM; truly bespoke devices might be very specific to a certain individual or sample, and so it could be more useful to re-shape the material into the best-fitting form rather than repurposing an existing part. However, some challenges serve to complicate the recycling of the 3D printed polymer parts used in electrochemistry.

The first challenge for recycling is that many polymers, including those commonly used for AM, are susceptible to ageing^21^ and deterioration in the conditions encountered in-service,^22,23^ as well as during recycling.^24^ In particular, poly(lactic acid) (PLA), one of the most commonly used polymers for FFF, is known to change physicochemical properties upon recycling. For example, Beltran et al. found that when PLA was recycled in a fashion typical of the plastics recovery industry, there were increases in polymer crystallinity accompanied by decreases in molecular weight and, subsequently, melt viscosity.^25^ Property changes, such as these can affect how the polymer behaves in subsequent reprinting; for example, Anderson observed that recycled PLA (rPLA) filaments were more likely to cause nozzle clogging and subsequent part defects in FFF.^26^ This said, there are recent examples of using rPLA for the production of conductive filaments to form the basis of energy storage devices^27^ and electroanalytical sensors.^28^ Due to the biodegradable nature of PLA, composting of the material is an option, however, this requires favorable environmental conditions and the presence of the appropriate enzymes.^29^ Additionally, more work needs to be done to appropriately assess the effect of the carbon fillers and plasticizers on the degradation process.

The second issue associated with recycling AM electrochemical devices specifically is the presence of conductive filler particles. When recycling these materials, there is no guarantee that the conductive filler will be uniformly distributed in the recycled material as that provided by the manufacturer, and this could affect its electrical properties. Additionally, there are also recent examples of electrochemical cells being produced in which conductive and non-conductive materials are combined into a single, inseparable part, for example, by the use of an FFF printer capable of dual-extrusion.^9^ Recycling such parts will produce materials, which are non-conductive but nonetheless contain a small amount of conducting fillers. This has the potential to change fundamental material properties. Additionally, if such a cell recycled repeatedly for use as a non-conductive cell wall material alongside a fresh conductive filament, the recycled cell wall material would, after sufficient recycling iterations, contain enough conductive fillers to short-circuit the whole device. It is therefore important to establish both the extent to which polymer deterioration limits the fundamental recyclability of AM electrochemical devices, and the extent to which recycling mixed-material electrochemical devices is feasible for the reproduction of the same quality of parts.

In this study, we show the successful recycling of the mixed-material electrochemical cells, as previously reported.^9^ The cells are processed to distribute the carbon black from the electrodes throughout the polymeric matrix before being repurposed into a non-conductive filament. This filament is shown to successfully act as the cell body in the same AM electrochemical cell print before being recycled a second time. Furthermore, we present how the original electrochemical cells can be processed with increased amounts of carbon black added to create a new conductive filament that outperforms the original commercial filament. The two recycled filaments can be used in partnership to create fully recycled electroanalytical cells that show enhanced electrochemical performance to the original cell. This presents a step-change in how AM and electrochemistry can be used together sustainably toward promoting circular economy electrochemistry.

## Experimental Section

### Materials

All chemicals used were of analytical grade and were used as received without any further purification. All solutions were prepared with deionized water of resistivity not less than 18.2 MΩ cm from a Milli-Q system (Merck, Gillingham, UK). Hexaamineruthenium(III) chloride (RuHex), acetaminophen (ACOP), sodium hydroxide, and phosphate buffered saline (PBS) tablets were purchased from Merck (Gillingham, UK). Potassium chloride and carbon black (Super P, 99%+) were purchased from Fisher Scientific (Loughborough, UK). The commercial non-conductive polylactic acid (PLA) filament used was Raise3D Premium PLA (1.75 mm, Raise3D, California, US), and the commercial conductive PLA filament used was a commercial carbon black/PLA filament (1.75 mm, Protopasta, Vancouver, Canada), both purchased from Farnell (Leeds, UK). All other filaments used were produced in-house, as outlined below. Real samples (effervescent tablets) Pandadol ActiFast soluble tablets (500 mg, GlaxoSmithKline, Middlesex, UK) were purchased from a local convenience store.

### Additive Manufacturing

All electroanalytical cells were produced using FFF on freshly calibrated Raise3D E2 independent dual extruder (IDEX) 3D-printers (Raise3D, California, US), as described in our previous study.^9^ Briefly, all designs and .3MF files were produced using Autodesk Fusion 360 and then sliced and converted to. GCODE files using the open-source software ideaMaker 4.0.1 (Raise3D, California, US). The cells were all printed using the appropriate non-conductive filament on the left nozzle (0.4 mm) at a set temperature of 210 °C, while the conductive carbon black/PLA was printed on the right nozzle (0.4 mm) at a set temperature of 220 °C. The printing bed temperature was set at 50 °C throughout the prints. All cells were printed using a layer height of 0.1 mm, a shell width of 1 mm, and 100% infill, the infill speed of 70 mm/s for the standard PLA profile, and 35 mm/s speed for the CB/PLA. This print had a purge block located close to the cells, as well as a skirt to help prime the nozzle prior to printing the first layer and between each extruder change.

Coupons for tensile testing were produced in accordance with a Type IV specimen from ASTM D638 using a Prusa i3 MK3S+ 3D-printer (Prusa Research, Prague, Czech Republic). Both commercial and recycled filaments were printed using a 0.4 mm nozzle at a temperature of 210 °C, with a layer height of 0.1 mm, infill of 100%, and a print speed of 35 mm/s.

### Filament Production

All polymer samples were dried in an oven at 60 °C for a minimum of 2.5 h to remove any residual water from the polymer and then granulated prior to filament extrusion using a Rapid Granulator 1528 (Rapid, Sweden). This sample was collected and added to the hopper of the EX6 extrusion line (Filabot, VA, USA), with the four heat zones set to 60, 190, 195, and 195 °C, respectively. The molten polymer strand was pulled along an Airpath cooling line (Filabot, VA, USA), through an inline measure (Mitutoyo, Japan) and collected on a Filabot spooler (Filabot, VA, USA).

### Print Recycling Process

Filament using only the granulation processing was produced as outlined above. For ball-milled samples, granulated AM cup cells (20 g) and Al_2_O_3_ grinding balls (6 × 20 mm Ø, 15 × 8 mm Ø) were placed inside a Al_2_O_3_ grinding jar and milled at 450 rpm for 60 min at ambient temperature using a PM700 planetary ball mill (Retsch, Germany). For thermomelt samples, granulated AM cup cells (60 g) were blended in a heated chamber (170 °C) with Banbury rotors at 70 rpm for 3 min using a Thermo Haake Poydrive dynameter fitted with a Thermo Haake Rheomix 600 (Thermo-Haake, Germany). The resultant sample was allowed to cool to room temperature before being granulated once more. For the solvent-mixed samples, granulated AM cup cells (105 g) were placed in a glass bottle fitted with a magnetic stirrer and dichloromethane (900 mL) was added. The bottle was sealed with a ventilation cap to limit evaporation while also preventing dangerous pressure build-up in the container. The mixture was then left to dissolve under magnetic stirring over several days at ambient temperature (approximately 18–20 °C). As indicated by level markers on the bottle, there was no significant degree of DCM evaporation from the capped bottle during this time. After the polymer had fully dissolved, the solution was poured into a glass tray and allowed to evaporate under fumehood extraction for 24 h. The cast polymer film was then removed from the tray and heated overnight in an oven at 80 °C to remove the residual solvent (with the oven fitted with extraction to remove harmful DCM vapors from the lab). The resultant sample was allowed to cool to room temperature before being granulated once more.

For the recycled conductive filament, the cup cells were dried and granulated as outlined above before being blended alongside additional carbon black (25 wt %) in a heated chamber (170 °C) with Banbury rotors at 70 rpm for 3 min using a Thermo Haake Poydrive dynameter fitted with a Thermo Haake Rheomix 600 (Thermo-Haake, Germany). This mix was allowed to cool before being granulated and added to the hopper of the EX6 extrusion line (Filabot, VA, USA), with the four heat zones set to 60, 190, 195, and 195 °C, respectively. The molten polymer strand was pulled along an Airpath cooling line (Filabot, VA, USA), through an inline measure (Mitutoyo, Japan), and collected on a Filabot spooler (Filabot, VA, USA).

### Electrochemistry

An Autolab PGSTAT128N potentiostat (Utrecht, the Netherlands) was used in conjunction with NOVA 2.1.5 (Utrecht, the Netherlands) to carry out electrochemical measurements using a three-electrode configuration. The additive manufacturing electrodes (AMEs) were used as the working, counter, and reference electrodes in all cases. All solutions were prepared using deionized water of resistivity not less than 18.2 MΩ cm from a Milli-Q system (Merck, Gillingham, UK). Solutions of 1.0 mM RuHex (0.1 M KCl) were degassed thoroughly for at least 15 min with nitrogen prior to any electrochemical measurement.

The activation of the AMEs was performed before all electrochemical experiments. This was achieved electrochemically in NaOH as described in the literature.^30^ Briefly, the AMEs were connected as the working electrode in conjunction with a nichrome wire coil counter and Ag|AgCl (3 M KCl) reference electrode and placed in a solution of NaOH (0.5 M). Chronoamperometry was used to activate the AMEs by applying a set voltage of +1.4 V for 200 s, followed by applying −1.0 V for 200 s. The AMEs were then thoroughly rinsed with deionized water and dried under compressed air before further use.

The heterogeneous electrochemical rate constants, *k*^0^, were calculated as an average from three sets of scan rate studies for each electrode. These utilized 10 different scan rates of 5, 10, 15, 25, 50, 75, 100, 150, 250, and 300 mV s^–1^, respectively. These were performed against the near ideal outer-sphere redox probe RuHex (1 mM in KCl) utilizing identical AMEs for the working, counter, and reference electrodes. The widely utilized Nicholson method^31^ was used for quasi-reversible electrochemical reactions through the following formula:
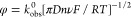
1where φ is a kinetic parameter, *D* is the diffusion coefficient for RuHex (*D* = 9.1 × 10^–6^ cm^2^ s^–1^),^32^*n* is the number of electrons that are taking part in the process, *F* is the faraday constant, ν is the scan rate, *R* is the gas constant, and *T* is the temperature in Kelvin. In order to calculate the HET rate constant, we use the peak to peak separation (Δ*E*_p_) to deduce φ, where Δ*E*_p_ is obtained at various voltammetric scan rates.^33^ The standard heterogeneous constant (*k*_obs_^0^) can be calculated via the gradient when plotting φ against [π*Dn*ν*F*/*RT*]^−1/2^. In cases where Δ*E*_p_ is bigger than 212 mV, the following equation should be implemented:
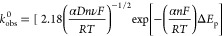
2where α is assumed to be 0.5.^34^

The electroactive area of the electrode, *A*_e_, is calculated using the Randles–Ševćik equation at non-standard conditions for quasi- eq 3 and irreversible eq 4 electrochemical processes when appropriate:^35^
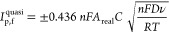
3
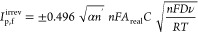
4where in all cases, *n* is the number of electrons in the electrochemical reaction, *I*_p,f_ is the voltammetric current (analytical signal) using the first peak of the electrochemical process, *F* is the Faraday constant (C mol^–1^), ν is the applied voltammetric scan rate (V s^–1^), *R* is the universal gas constant, *T* is the temperature in Kelvin, *A*_real_ is the electroactive area of the electrode (cm^2^), *D* is the diffusion coefficient (cm^2^ s^–1^), and α is the transfer coefficient (usually assumed to be close to 0.5). It should be noted that for eqs 2 and 3 to hold that the electrode should be flat and non-porous, whereas AMEs are made from different carbons, polymers, and plasticizers. However, the surface roughness of AMEs after printing and activation remain in the region of the hundreds of nanometers,^18^ and therefore, over the timescale of a voltammetric experiment, the diffusion layer is larger than the AME micro-features of the carbon black, meaning the equations hold.^36,37^

### Physiochemical Characterization

Melt flow analysis was conducted following ASTM D1238 using a Ray Ran Melt Flow indexer (Nuneaton, UK). A 4.00 g sample of polymer was added to the heating chamber at 190 °C and heated for 3 min under a 2.16 kg weight. After 3 min, the polymer runoff was cut and allowed to flow for 30 s before being cut again and this sample was weighed; the weight was multiplied by 20 to give a melt flow index (MFI) in g/10 min. This process was repeated, and an average value was taken of the melt-flow indexes.

Scanning electron microscopy (SEM) measurements were recorded on a Supra 40VP Field Emission (Carl Zeiss Ltd., Cambridge, UK) with an average chamber and gun vacuum of 1.3 × 10^–5^ and 1 × 10^–9^ mbar, respectively. Samples were mounted onto aluminum SEM pin stubs (12 mm diameter, Agar Scientific, Essex, UK), and a thin layer of Au/Pd (8 V, 30 s) was sputtered onto the electrodes using a SCP7640 coater (Polaron, Hertfordshire, UK).

Thermogravimetric analysis (TGA) was performed using a Discovery Series SDT 650 controlled by Trios Software (TA Instruments, DA, USA). Samples were mounted in alumina pans (90 μL) and tested using a ramp profile (10 °C min^–1^) from 0 to 800 °C under N_2_ (100 mL min^–1^).

X-ray photoelectron spectroscopy (XPS) data were acquired using an AXIS Supra (Kratos, UK), equipped with a monochromated Al X-ray source (1486.6 eV) operating at 225 W and a hemispherical sector analyzer. It was operated in a fixed transmission mode with a pass energy of 160 eV for survey scans and 20 eV for region scans with the collimator operating in the slot mode for an analysis area of approximately 700 × 300 μm; the FWHM of the Ag 3d5/2 peak using a pass energy of 20 eV was 0.613 eV. Before analysis, each sample was ultrasonicated for 15 min in propan-2-ol and then dried for 2.5 h at 65 °C, as this has been shown in our unpublished data to remove excess contamination from PLA and therefore minimize the risk of misleading data. The binding energy scale was calibrated by setting the carbon–carbon sp^3^ C 1s peak to 285 eV. This calibration is acknowledged to be flawed,^38^ but it was nonetheless used in the absence of reasonable alternatives and because only limited information was to be inferred from absolute peak positions.

Changes in the mechanical performance of the recycled filament were performed through tensile testing of 3D-printed parts using a Hounsfield H10KS. Tensile testing was carried out in accordance with ASTM D638, specifically using Type IV specimen dimensions and a testing rate of 5 mm min^–1^. Cross-sectional areas used to determine ultimate tensile strength were calculated using the average width and thickness measurements taken from three points along the gauge length of each test coupon.

## Results and Discussion

The number of publications combining AM and electrochemistry has significantly increased in the last 5 years.^39^ As such, key advancements in the field have been made, such as printing cells with embedded electrodes in a single, mixed-material print.^9,40^ These prints provide the opportunity for users to print sensors directly, with no need for post-print assembly. However, this printing methodology introduces significant limitations when it comes to recycling these devices as there is a mixture of filaments, fillers, and plasticizers all in a single print. To overcome this issue, recycle these devices and align AM with the circular economy concept and sustainability goals established by the United Nations,^41^ development of a processing method for mixed-material devices like these is necessary. Herein, we look to evaluate different processing possibilities to produce high-quality end products.

### Development of Non-Conductive Filament from Recycled Prints

The production of recycled non-conductive filament from old mixed-material prints is an engineering challenge due to the different concentrations of fillers in each component of the print. In this study, all prints had previously been utilized for electrochemical experiments,^9^ with the body of the cells printed from standard commercial PLA and the electrodes printed from commercially purchased PLA/carbon black (PLA/CB) filament. All prints were thoroughly washed with deionized water and dried to remove any potential contaminants. To create a sealed container for electrochemical studies, the electrodes were embedded into the design and printed on an independent dual-extruder 3D printer. Therefore, to recycle these cells in addition to standard PLA, the conductive filament that incorporates PLA (>65 wt %) carbon black (<21.43 wt %) and an unnamed plasticizer (<12.7 wt %) must be accounted for. In the design of this electrochemical cell, 37.6 g of non-conductive PLA and 0.9 g of conductive PLA/CB were utilized, meaning that the amount of carbon black in the new filament should theoretically be equal to ∼0.5 wt %. This does not reach the levels required to induce conductivity in the filament, meaning that with proper dispersion throughout the polymer matrix, it should be suitable to make a new non-conductive filament.^42^

**Figure 1:**
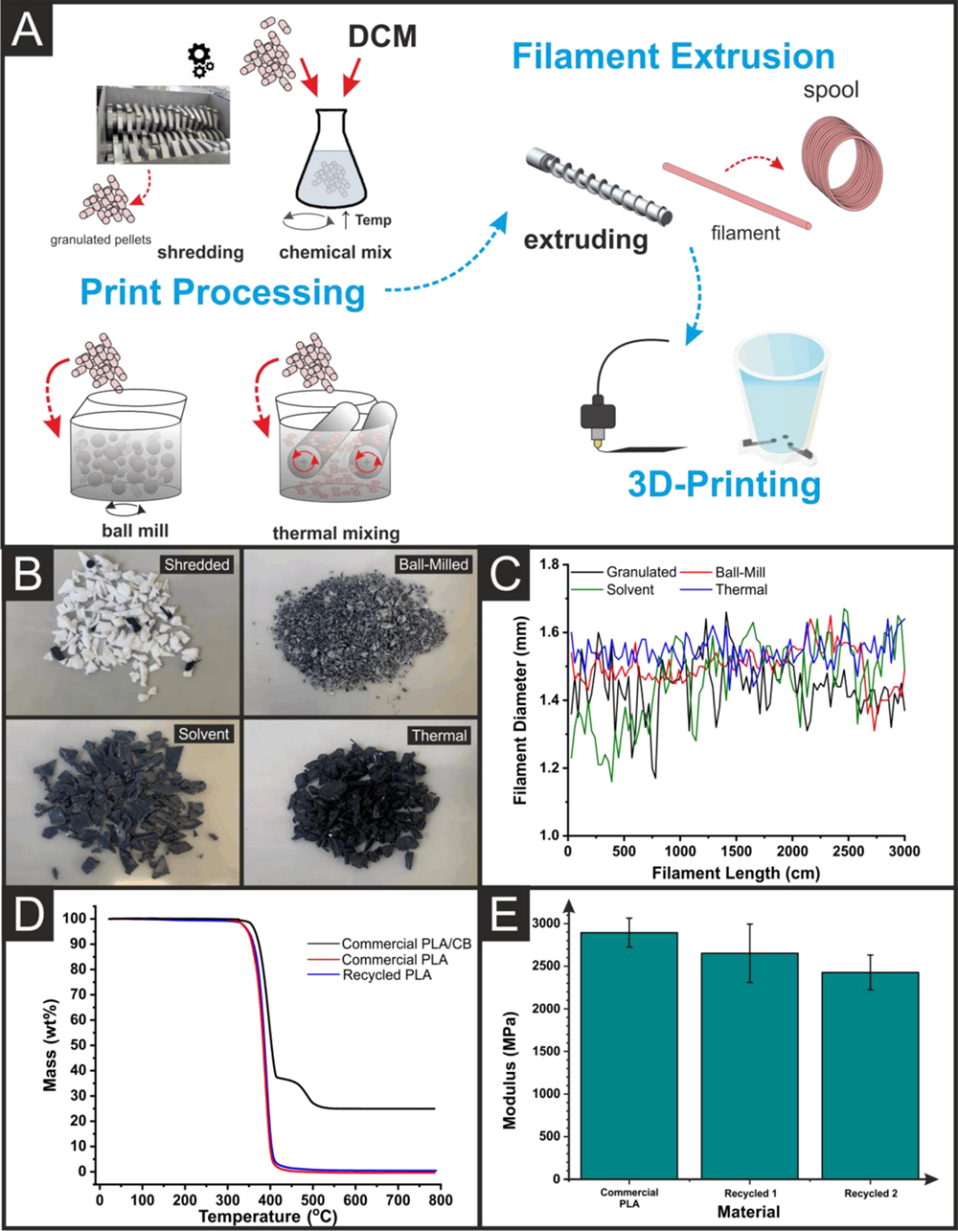
(A) Schematic of the four recycling processes used to feed into 3D-printing filament production. (B) Photographs of the shredded polymer collected after each of the recycling processes used. (C) Plot of the measured filament diameter over an arbitrary portion of the length of the filament for the four different recycling processes. (D) Thermogravimetric analysis of the recycled filament produced using thermal mixing versus the commercial non-conductive and conductive filaments used to print the original cell. (E) Plot of the average Young’s moduli for parts made using commercial non-conductive PLA versus two cycles of the recycled filament (the uncertainty is the standard deviation from five repeat samples).

Four different processing methods were tested to reconstitute a non-conductive filament from the printed parts, Figure 1A, including granulation, ball-milling, solvent mixing, and thermal mixing. It is important to note that all prints had been rinsed with deionized water and then fully dried in an oven at 65 °C for 2.5 h before processing. All methods began with passing the old electrochemical cells through a granulator and collecting the subsequent granulated mix. For the granulation method, this mix was passed straight through the extruder to create the filament. The ball-milling method passed the granulated mix through a ball mill at 450 rpm for 1 h. at ambient temperature, and the powdered plastic was then collected. The solvent mixing method involved dissolving of the granulated mix in dichloromethane (DCM), before casting the resultant solution in a dish to evaporate the residual solvent, similar to methods reported in the literature.^43,44^ The thermal method involved mixing the granulated sample at 170 °C and 70 rpm for 3 min, providing enough mixing to disperse the carbon black throughout the polymer matrix, while minimizing exposure to increased thermal stress. All of these methods were passed through the granulator once more before extrusion. Figure 1B presents images of the different mixes obtained through each method, which were then passed through the extruder to produce the new non-conductive filament. It can be seen that there are significant differences between the samples, with both the granulated and ball mill samples consisting of separate white and black particles (corresponding to particles enriched with PLA or PLA/CB, respectively), and both the solvent and thermal mixing methods producing samples with a more even color distribution, which is likely an indication of more even distribution of the carbon black within the polymer. Figure 1C presents the measured diameter of each produced filament as a function of length, highlighting how the thermal mixing method produced a filament with a more consistent form. The thermal-mixing method produced the best results with a diameter relative standard deviation (RSD) along the filament length of RSD = 3.22%, due to the excellent dispersion of the conductive material through the polymer matrix. The solvent method produced the largest amount of deviation in the filament diameter with RSD = 8.61%, which is speculated to be due to inhomogeneities caused by the solvent casting process (e.g., the presence of bubbles) rather than the distribution of fillers within the material, since as discussed this sample appeared visually to have a more homogenous distribution of carbon black than the granulated and ball mill samples, which both had more consistent filament diameter. Granulated (RSD = 5.67%) and ball-milling (RSD = 4.13%) were next, where there was minimal mixing of the conductive filament into the non-conductive filament, which was also seen in the ball-milled sample but to a lesser extent. The improved filament production from the thermal mixing methods is attributed to the more even dispersion of CB particles throughout the PLA matrix, presumably due to the higher shear rates being applied throughout the molten polymer using the rheomixer. This reduced the amount of blocking at the die head significantly, resulting in a more even flow of polymer melt.

Each of these filaments was then characterized through melt flow indexing and using TGA, with the results summarized in Table 1. Example TGA data for one sample obtained by the thermal mixing method plotted alongside examples of commercial conductive and non-conductive PLAs is presented in Figure 1D. It can be seen that all of the filaments produced a similar onset temperature of degradation of 307–309 °C, which is in good agreement with commercially purchased non-conductive and conductive PLA measured previously.^28^ From the TGA data, it was possible to calculate the filler content in the samples, specifically from the mass remaining at the end of the measurement. The results were recorded as the average of three samples, which were taken from different, random areas of the filament. It can be seen that the thermal mixing methodology produced the filler content closest to the expected theoretical content of the filler and produced the lowest deviation in the measurements. This indicated that the conductive filler was more evenly dispersed throughout the polymer matrix. The MFI measures the flow of a thermoplastic polymer melt over time and is an indirect measurement of melt viscosity and hence the molecular weight.^45^ An increase in this value indicates a lowering of the polymer molecular weight, in this case through chain scission caused by either thermal, mechanical, or chemical degradation.^46,47^ It can be seen that the granulated sample produces the lowest MFI, expected as all the samples went through this process, with the thermal and solvent mixing methods producing similar results and the ball-milling causing significantly more polymer degradation. These tests confirmed that the thermal mixing method produced the best combination of results in terms of filament quality and filler dispersion, while minimizing the polymer degradation, and was therefore chosen as the recycling method for use throughout the rest of this study.

**Table 1:** Thermal Properties of the Recycled Filament Produced by Different Processing Methods^a^

production method	melt flow index (g/10 min)	onset temperature (°C)	filler content (wt %)
granulation	5.73 ± 0.49	307 ± 2	1.71 ± 0.94
ball-milling	10.2 ± 0.3	309 ± 3	2.15 ± 0.32
solvent mixing	6.99 ± 0.32	308 ± 2	2.49 ± 1.06
thermal mixing	7.16 ± 0.36	307 ± 5	0.67 ± 0.31

a Summarizing the melt flow index, the degradation onset temperature, and the conductive filler (carbon black) content. The uncertainties in melt flow index, onset temperature, and filler content are the standard deviations of three repeat measurements.

To test this recycling methodology toward use in an ideal circular economy, where discarded objects can be continually reused, the recycled filament was used to print the same electroanalytical sensing platforms originally used. These were then subjected to the same recycling process to produce a second iteration non-conductive filament and again to create a third. All of the compositions were tested for their MFI, Table 2, after processing but before extrusion into a filament, with significant increases seen from 7.16 ± 0.36 to 29.6 ± 0.8 g/10 min for the first and third iterations, respectively. The TGA data obtained for the produced filaments, along with a comparison to the commercial PLA filament is shown in Table 2. It should be noted that the levels of degradation seen in the third iteration were too high for a printable filament to be produced and therefore they could not be analyzed. It can be seen that the onset temperature of degradation over the two iterations shows excellent agreement, and that the filler content of the second iteration has increased to 1.71 ± 0.84 wt %. Dogbones 3D printed from commercial PLA and two recycled PLA filaments were then subjected to tensile testing. Figure 1E shows the average Young’s moduli obtained for five repeat measurements, and it can be seen that there is a decrease in the calculated modulus for each recycling iteration, but nonetheless, a significant proportion of the initial stiffness is maintained. Overall, the above results demonstrate that old additively manufactured mixed material prints can indeed be recycled into non-conductive filament to make parts retaining most of their original properties, but to recreate a new fully recycled electroanalytical sensing platform, a conductive recycled filament must also be produced.

**Table 2:** Thermal Properties of the Commercial Filament, and the Recycled Filament Produced by Thermal Mixing over Different Cycles^a^

non-conductive iteration	melt flow index (g/10 min)	onset temperature (°C)	filler content (wt %)
commercial PLA	4.16 ± 0.04	305 ± 5	0
iteration 1	7.16 ± 0.36	307 ± 5	0.67 ± 0.31
iteration 2	17.4 ± 0.1	307 ± 1	1.71 ± 0.84
iteration 3	29.6 ± 0.8	N/A	N/A

a Summarizing the melt flow index, the degradation onset temperature, and the conductive filler (carbon black) content. The uncertainties in melt flow index, onset temperature, and filler content are the standard deviations of three repeat measurements.

### Production and Characterization of Conductive Filament from Recycled Prints

**Figure 2:**
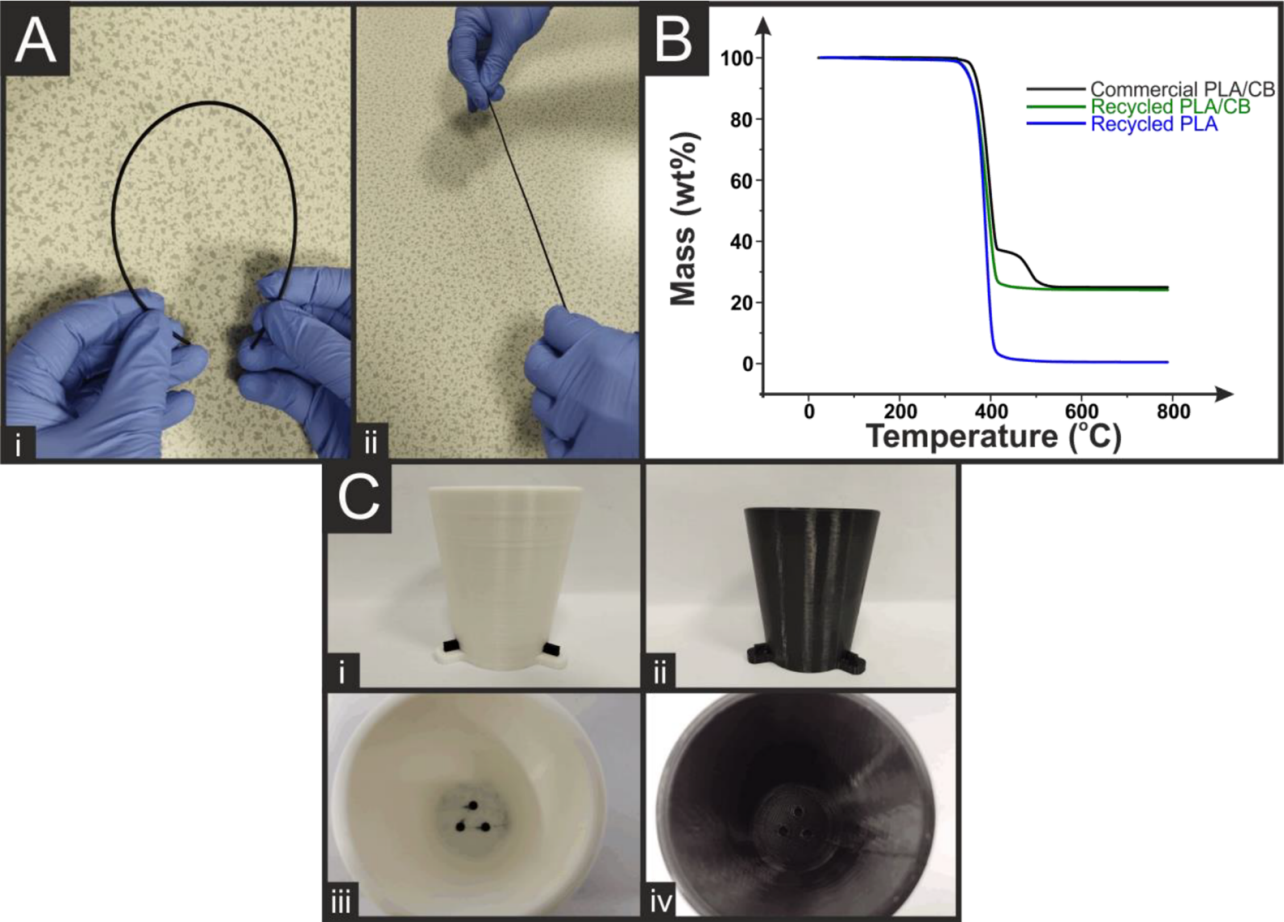
(A) Photographs of the recycled conductive filament highlighting its flexibility through (i) bending the filament and (ii) straightening the filament. (B) Thermogravimetric analysis of the recycled conductive filament against the recycled non-conductive filament and the commercially purchased conductive filament. (C) Photographs of the original and recycled 3D-printed electroanalytical cells, showing side views of the cup and connections (i, ii) and the top view (iii, iv) showing the embedded AMEs.

The recycled conductive filament was produced in a similar way to that above, but with the addition of carbon black to the thermal mixing chamber along with the recycled material to increase the amount of conductive fillers back to the level seen in the commercial conductive filament. This mixed composite was then granulated and passed through the extruder in the same way outlined above to produce a conductive filament with excellent flexibility, Figure 2A. TGA analysis, Figure 2B, confirmed that the recycled conductive filament had a similar filler content to the original commercially purchased conductive filament, 21 ± 2 wt % compared to 21 ± 3 wt %. It can be seen that there is no longer a second transition for the recycled conductive filament. This is because no additional plasticizer is added to the recycled filament, meaning theoretically there is under 0.1 wt % plasticizer in the final filament, compared to approximately 12.7 wt % in the as-provided commercial conductive filament. This recycled conductive filament was then used in conjunction with the recycled non-conductive filament described earlier to reproduce the electroanalytical sensing platform originally reported.^9^Figure 2C shows photographs of the original cell and fully recycled cell from the side and top views. Although harder to see due to both recycled filaments being black, the electrodes are still well defined with the recycled conductive filament having a matte finish compared to the gloss of the recycled non-conductive filament, which is due to the significantly increased CB filler content reducing the reflectivity of the material.

**Figure 3:**
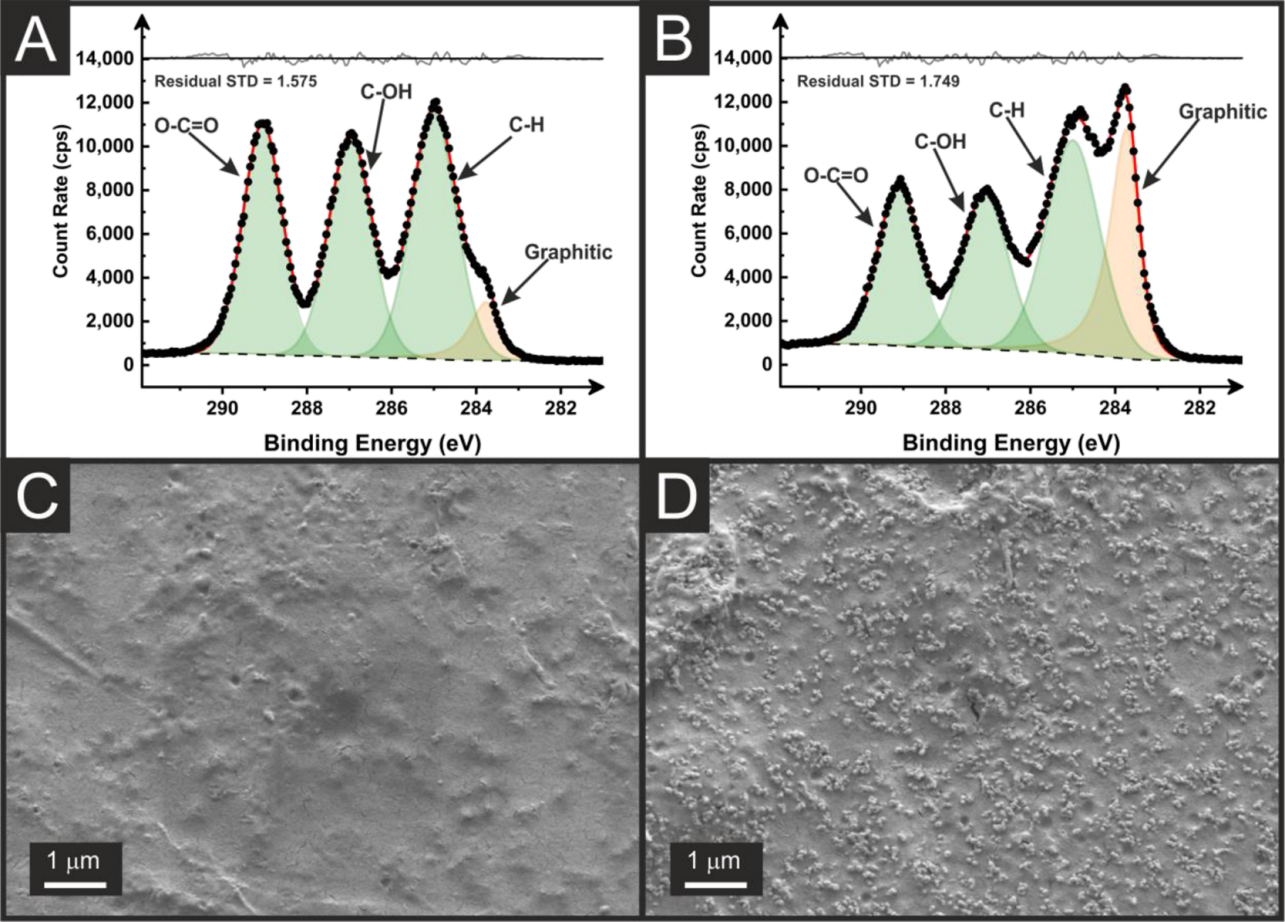
XPS C 1s spectrum for the rAMEs before (A) and after (B) electrochemical activation. SEM images of the rAMEs surface before (C) and after (D) electrochemical activation.

To investigate the surface chemical composition of the recycled additively manufactured electrodes (rAMEs) and confirm the success of electrochemical activation, XPS analysis was performed. Figure 3A,B shows the C 1s environment for the as-printed rAME and electrochemically activated rAME, respectively. Electrochemical activation was performed using chronoamperometry in a sodium hydroxide (0.5 M), which removes PLA from the part surface, as seen previously in the literature.^30^ Before activation, Figure 3A, the C 1s environment shows a spectrum similar to that of PLA, with three peaks of similar intensity corresponding to the three carbon environments in the PLA chain. There is also a small shoulder peak visible, which is fitted with an asymmetric peak consistent with the X-ray photoelectron emission by graphitic carbon,^48,49^ which suggests that there is more carbon black available near the surface of the AME compared to XPS seen for the commercial filament in previous study.^9,18,28^ This is proposed to be because of the significantly reduced plasticizer content of the filament, as it has been seen that the plasticizer can migrate to the surface of the print layer which would be expected to obscure observation by XPS of the carbon filler until after activation.^27^ Upon activation, Figure 3B, there is a significant increase in the intensity of the asymmetric graphitic carbon peak, indicating that significant amounts of the base PLA of the rAME have been removed, revealing larger amounts of the conductive carbon black filler. This is confirmed when comparing the SEM images of the rAME surfaces before and after activation, Figure 3C,D. It can be seen that before activation, Figure 3C, there is some visible carbon black with a large amount of smooth PLA plastic coating. After electrochemical activation, this smooth PLA coating is significantly stripped from the surface leaving a significantly increased concentration of carbon black.

### Electrochemical and Electroanalytical Characterization of the Recycled Filament

For electrochemical testing, three varieties of sensing platforms were printed: the first one using commercial non-conductive and conductive filaments, the second one using recycled non-conductive filament and commercial conductive filament, and the third one using recycled filament for both the casing and the AMEs. Prior to electrochemical characterization, the resistance along the connection length of the AMEs was measured using a multi-meter as this has been shown to affect the electrochemical performance of AMEs.^50^ Both cells using the commercial conductive filament obtained similar resistance values of 1.15 ± 0.17 and 1.17 ± 0.13 kΩ, which was expected, as they are printed from the same material. The AME printed from the recycled conductive material had a significantly lower measured resistance of 0.68 ± 0.09 kΩ across the connection length, which is attributed to the increased concentration of conductive filler seen on the surface of the rAME, as seen in the XPS and SEM characterization.

**Figure 4:**
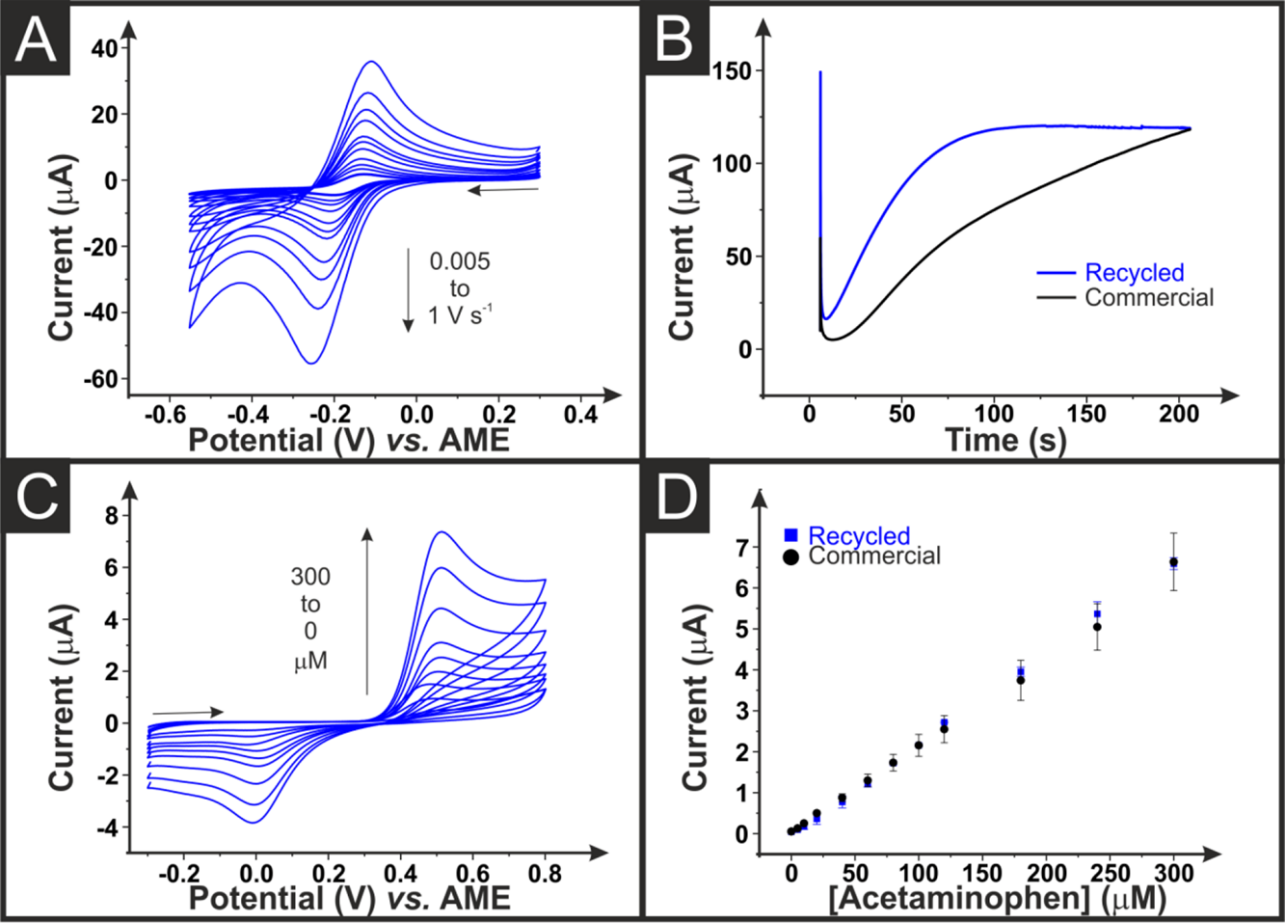
(A) Scan rate study (0.005–1 V s^–1^) for an AME printed from the recycled conductive filament. Performed in hexaamineruthenium(III) chloride (1 mM, 0.1 M KCl) with an AM counter and a reference electrode. (B) Chronoamperometric activation profiles for the application of +1.4 V (vs AME) for 200 s to AMEs printed from commercial (black) and recycled (blue) conductive filament. Performed in NaOH (0.5 M) with an AM counter and reference electrode. (C) Cyclic voltammograms (ν = 0.05 V s^–1^) for the determination of acetaminophen (0–300 μM, 0.01 M PBS at pH = 7.4) using an AME printed from the recycled conductive filament against a 3D-printed counter and reference electrode. (D) Analytical curves for the detection of acetaminophen using AMEs printed from the commercial (black) and recycled (blue) conductive filament.

Initial electrochemical characterization of the three cells was performed using scan rate studies on as-printed electrodes, Figure 4A, against the near-ideal outer sphere redox probe hexaamineruthenium(III) chloride (RuHex, 1 mM in 0.1 M KCl), as this allows for the best determination of the heterogeneous electrochemical rate constant (*k*^0^) and the real electrochemical surface area (*A*_e_).^32^ The data obtained from the electrochemical and electroanalytical studies in this study is summarized in Table 3. It can be seen that the cell containing the rAME gives an enhanced *k*^0^ of (3.6 ± 0.4) × 10^–3^ cm s^–1^ compared to the commercial AMEs of (2.5 ± 0.2) × 10^–3^ cm s^–1^ and (2.9 ± 0.6) × 10^–3^ cm s^–1^. Additionally, the rAME shows an increased electrochemical surface area of 0.081 ± 0.001 cm^2^ compared to 0.075 ± 0.001 and 0.074 ± 0.002 cm^2^. This increase in electrochemical area is in agreement with the XPS and SEM data obtained earlier, whereby an increased amount of graphitic carbon was seen on the surface of non-activated rAMEs compared to standard commercial ones, as seen in other studies.^18,28^ This data shows that the recycled conductive filament gives excellent electrochemical performance compared to the commercial equivalent.

**Table 3:** Electrochemical and Electroanalytical Properties of the Different Cell Iterations^a^

non-conductive filament	conductive filament	AME resistance (kΩ)	*k*^0^ (×10^–3^ cm s^–1^)	*A*_e_ (cm^2^)	sensitivity (μA μM^–1^)	LOD (μM)	recovery (%)
commercial	commercial	1.15 ± 0.17	2.5 ± 0.2	0.075 ± 0.01	22.3 ± 0.7	4.5 ± 0.9	97 ± 5
recycled - 1	commercial	1.17 ± 0.13	2.9 ± 0.6	0.074 ± 0.02	21.5 ± 0.5	4.2 ± 1.2	99 ± 6
recycled - 1	recycled	0.68 ± 0.09	3.6 ± 0.4	0.081 ± 0.01	22.4 ± 0.2	3.2 ± 0.8	95 ± 5

a Highlighting the resistance across the AME connection length, the heterogeneous electrochemical rate constant (*k*^0^), the real electrochemical surface area (*A*_e_), sensitivity for acetaminophen determination, a limit of detection for acetaminophen, and the recovery % from a real pharmaceutical sample. The uncertainties in the AME resistances are the standard deviation of 12 AMEs. The uncertainties in the *k*^0^, *A*_e_, sensitivity, LOD, and recoveries are the standard deviations across three different AME measurements.

Electrochemical activation of AMEs has been shown to significantly improve the performance of AMEs toward inner sphere molecules.^30^ Therefore for the determination of acetaminophen, electrochemical activation was performed on the AMEs, as seen in previous study.^9^ The electrochemical activation profiles for the rAME and commercial AME are presented in Figure 4B. Both AMEs reach a similar current value by the end of the 200 s activation at ∼120 μA, indicating a similar level of conductive filler being available for electrochemical reactions after activation. It is theorized that the rAME reaches this current level quicker due to the lower resistance of the recycled filament.

Once activated, the electroanalytical cells were used for the detection of acetaminophen using cyclic voltammetry (ν = 0.05 V s^–1^). An example of the obtained voltammograms using the rAMEs is presented in Figure 4C, where there is a clear peak for the two-electron oxidation of acetaminophen to *N*-acetyl-*p*-benzoquinone-imine^9^ at ∼ +0.5 V vs a 3D-printed *pseudo*-reference electrode, followed by a smaller reduction peak at ∼0.0 V. Acetaminophen (5–300 μM) was added to PBS (pH = 7.4), and the peak oxidation current was obtained for all three systems. Figure 4D shows the linear calibration plots for the two cells using recycled non-conductive filament (the data for the original cell is shown within Table 3), with the black data corresponding to the commercial AME and the blue data corresponding to the rAME. It can be seen that both systems show good agreement and similar electroanalytical characteristics, with the rAME exhibiting a sensitivity and a limit of detection (LOD) of 22.4 ± 0.2 μA μM^–1^ and 3.2 ± 0.8 μM, respectively. This is a slight improvement on the commercial AME, which exhibited values of 21.5 ± 0.5 μA μM^–1^ and 4.5 ± 1.2 μM, respectively. All three systems were then tested toward the detection of acetaminophen in a real pharmaceutical sample, where they all exhibited excellent recovery values between 95 and 99% against the UV/vis data obtained previously.^9^

This study presents a paradigm shift in how AM can be used more sustainably in electrochemical research by recycling mixed-material prints. This can be achieved as a non-conductive filament to be used in future prints, or even to produce new conductive filament which can match or even exceed the performance of the original commercial conductive filament. We expect this study to encourage more research into how AM (specifically FFF) can improve the sustainability of electrochemical work due to its low-waste nature and ability to use recycled feedstocks.

## Conclusions

This study describes the recycling of additively manufactured electroanalytical sensing platforms printed from mixed-materials, including conductive fillers, into two new FFF 3D-printing filaments (one non-conductive and one conductive). Four processing methodologies (granulation, ball-milling, solvent mixing, and thermal mixing) were explored for the recycling of old electroanalytical cells into a new non-conductive filament, with thermal mixing producing the highest quality filament due to the even dispersion of conductive fillers throughout the polymer composite. Using this method, it was possible to print, use, and recycle the electroanalytical sensing platforms through two additional cycles, without adding in any additional material, before polymer failure. The recycled conductive filament was produced by incorporating additional carbon black into the polymeric mix to match the conductive filler content of the original commercial filament. This conductive filament was flexible and printed easily in combination with the recycled non-conductive filament. It was shown to have lower resistance than the original commercial conductive filament when measured in the final printed electrochemical cell and gave enhanced electrochemical performance against the near-ideal outer sphere redox probe hexaamineruthenium(III) chloride. When utilized for the determination of acetaminophen, the fully recycled electroanalytical cell matched the performance of the original produced from commercial filaments achieving a sensitivity of 22.4 ± 0.2 μA μM^–1^, a limit of detection of 3.2 ± 0.8 μM and a recovery value of 95 ± 5% when tested using a real pharmaceutical sample. This study represents a paradigm shift in how sustainability and recycling can be utilized within additively manufactured electrochemistry.
